# Functional and numerical responses of shrews to competition vary with mouse density

**DOI:** 10.1371/journal.pone.0189471

**Published:** 2018-01-03

**Authors:** Carolyn A. Eckrich, Elizabeth A. Flaherty, Merav Ben-David

**Affiliations:** 1 Oregon Department of Fish and Wildlife, La Grande, OR, United States of America; 2 Department of Forestry and Natural Resources, Purdue University, West Lafayette, IN, United States of America; 3 Department of Zoology and Physiology and Program in Ecology, University of Wyoming, Laramie, WY, United States of America; University of Missouri Kansas City, UNITED STATES

## Abstract

For decades, ecologists have debated the importance of biotic interactions (e.g., competition) and abiotic factors in regulating populations. Competition can influence patterns of distribution, abundance, and resource use in many systems but remains difficult to measure. We quantified competition between two sympatric small mammals, Keen’s mice (*Peromyscus keeni*) and dusky shrews (*Sorex monticolus*), in four habitat types on Prince of Wales Island in Southeast Alaska. We related shrew density to that of mice using standardized regression models while accounting for habitat variables in each year from 2010–2012, during which mice populations peaked (2011) and then crashed (2012). Additionally, we measured dietary overlap and segregation using stable isotope analysis and kernel utilization densities and estimated the change in whole community energy consumption among years. We observed an increase in densities of dusky shrews after mice populations crashed in 2012 as expected under competitive release. In addition, competition coefficients revealed that the influence of Keen’s mice was dependent on their density. Also in 2012, shrew diets shifted, indicating that they were able to exploit resources previously used by mice. Nonetheless, increases in shrew numbers only partially compensated for the community energy consumption because, as insectivores, they are unlikely to utilize all food types consumed by their competitors. In pre-commercially thinned stands, which exhibit higher diversity of resources compared to other habitat types, shrew populations were less affected by changes in mice densities. These spatially and temporally variable interactions between unlikely competitors, observed in a relatively simple, high-latitude island ecosystem, highlight the difficulty in assessing the role of biotic factors in structuring communities.

## Introduction

The relative importance of biotic interactions and abiotic factors in regulating population dynamics and community structure has been at the core of ecological investigations for over a century [1,2]. Ideas such as divergence of sympatric species [3,4], fundamental and realized niches [5], the neutral theory of biodiversity and biogeography [6], and compensatory ecological (or zero-sum) dynamics [7,8], all derive from a vast body of literature documenting the effects of biotic interactions (largely competition) on community composition, diversity, and species abundance. Nonetheless, recent re-analyses of published works suggest that in a majority of cases evaluated, abiotic factors may override the effects of biotic interactions [9].

Theory suggests that in cases with substantial overlap of fundamental niches (i.e., the set of resources potentially used by a species) and asymmetric competition, the superior competitor may drive the other species to local extinction [10,11]. However, when niche overlap is partial, the weaker competitor may be restricted to a portion of the space, which will represent its realized niche [5]. To allow coexistence of potential competitors, the realized niches of sympatric species may separate in three dimensions: time, space, and food [12,13]. For example, in Arizona, Brown [14] found that the coexistence of desert granivorous rodents was best explained by seasonal rotation in foraging strategies, where each species maximized its efficiency during a different part of the year. In such instances, competitive release could result in expansion of the realized niche of the weaker competitor [15].

Numerous empirical studies have shown that competitive interactions among species influence spatial and temporal patterns of distribution, abundance, and resource use [16,17]. Yet interspecific competition is difficult to measure in the field where multiple processes can interact to mask its effects. Most prominent is the impact of predation, which may produce apparent competition through indirect effects [18] or may conceal competitive interactions of prey populations [19]. In a meta-analysis, Gurevitch et al. [20] concluded that the presence of predators lessened the effect of competition. Further, competition may be obscured by unrelated evolutionary pressures. For example, Ben-David et al. [21] demonstrated that coastal river otters (*Lontra canadensis*) and American mink (*Neovison vison*) spatially segregate and coexist in the marine environment largely because of limitations on diving abilities of mink, potentially concealing past or ongoing competition [22]. Further, documenting biotic interactions on islands can be complicated by island size [23]. Thus, the role of interspecific competition in observed spatial and temporal partitioning is not always clear [24,25].

Small mammals (<1kg) represent a widespread and diverse assemblage of animals that are particularly suited for experimental study of competition [26,27]. For instance, removal and exclusion of the larger *D*. *heermanni* in California increased the numbers of the federally endangered *D*. *nitratoides nitratoides* by 500% within 12 months [28]. However, even experimental studies can fail to demonstrate the effects of competition when abiotic factors overshadow subtle biotic interactions [29]. As an alternative, negative correlations between the abundance of sympatric species (i.e., negative covariances) have been used to infer interspecific competition [13,16]. In such analyses, two sympatric species with similar ecological niches will likely exhibit higher competition coefficients (α) than a comparable pair exhibiting niche separation [30]. Luo et al. [31] found that when abundance was low and the velvet-furred rat (*Rattus lutreolus velutinus*) and the long-tailed mouse (*Pseudomys higginsi*) could segregate spatially, their α coefficients were small. During breeding and dispersal, when abundant populations precluded spatial segregation, their α coefficients were higher.

Populations of Keen’s mice (*Peromyscus keeni*) and dusky shrews (*Sorex monticolus*) are widely distributed across many of the islands in the Tongass National Forest (TNF) in Southeast Alaska [32]. Keen’s mice are a medium-sized *Peromyscus* sp. (18–24 g) considered habitat and dietary generalists [33,34]. Dusky shrews (6–8 g) are found in a variety of habitats and are primarily insectivores but are also known to consume mushrooms and lichens [35,36]. Both species are mostly nocturnal and populations fluctuate annually with mice usually reaching higher densities than shrews [37]. Although not commonly considered competitors, these species comprise the base of the food web in the coastal rainforests of Southeast Alaska and have the potential to influence each other’s vital rates through competitive interactions.

Our objective was to quantify competition between mice and shrews in the spatially heterogeneous landscape of the TNF. Because previous studies of Keen’s mice have shown that these small mammals exhibit variable dynamics in different seral stages of regenerating forests [33], we compared competitive interactions among different habitat types in our study area. We hypothesized that due to their larger body size and generalist foraging strategies, Keen’s mice would be a stronger competitor, exerting negative effects on the abundance of dusky shrews. We evaluated annual interspecific competition using the standardized regression technique [38] in 2010–2012. We hypothesized that coexistence of mice and shrews would be largely facilitated through partitioning of food resources and quantified dietary overlap and segregation between the two species using stable isotope analysis [39]. Finally, because theory predicts that whole community energy consumption will remain relatively static when interspecific competition alters species abundances (zero-sum dynamics; [40]), we predicted that shrew numbers would increase to energetically compensate for the competitive release from mice.

## Materials and methods

### Study area

**Fig 1:**
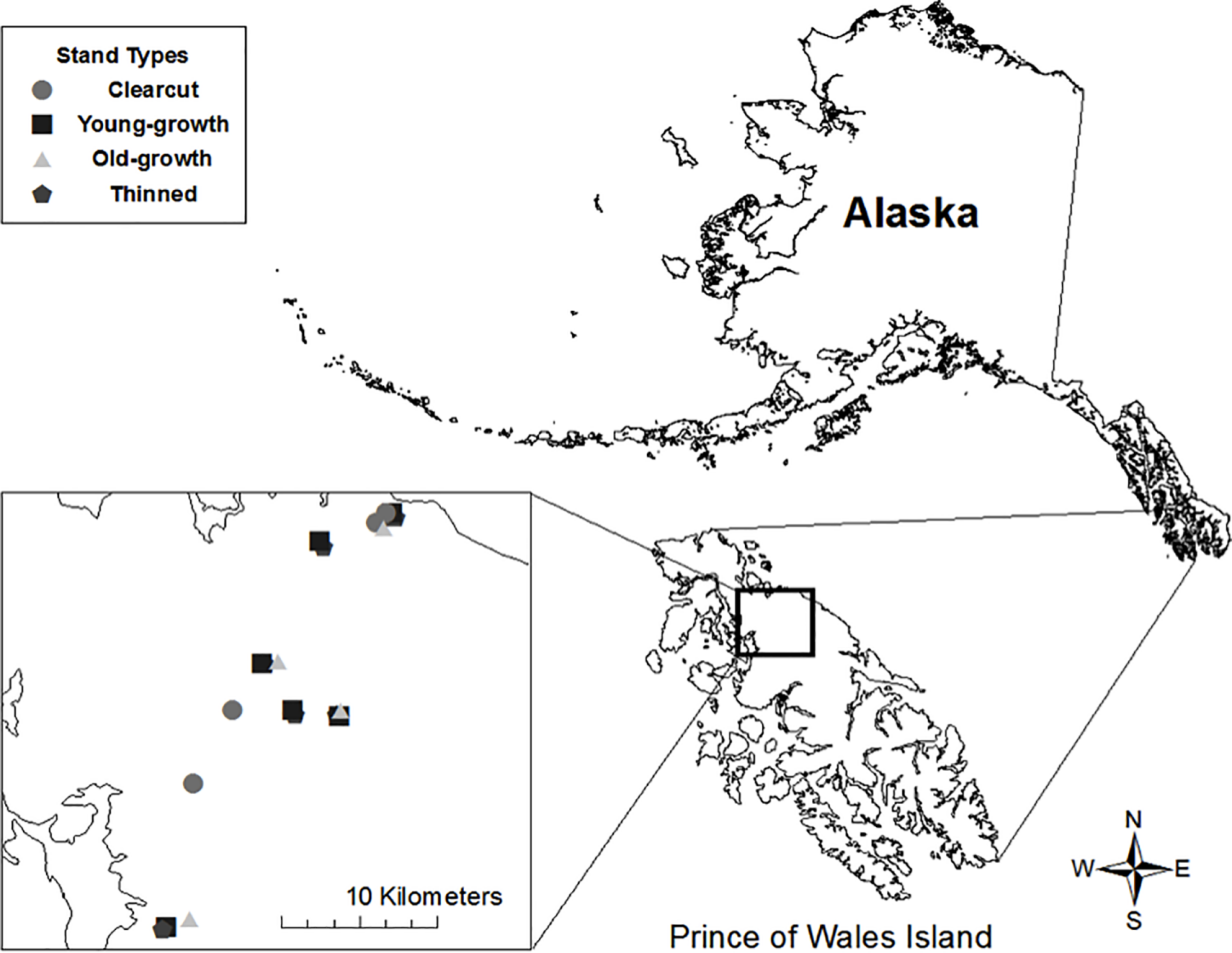
Study area and small mammal trapping grid locations on Prince of Wales Island, Alaska, USA, 2010–2012. Trapping grids were located in seven thinned, six un-thinned young-growth, four old-growth and four clearcut stands.

The TNF encompasses most of the Alexander Archipelago, a naturally fragmented system of large and small islands (Fig 1). Since the 1950’s, large-scale logging created over 174,000 ha of young stands in the TNF [41]. In 2001, the Tongass-Wide Young-Growth Studies (TWYGS) were initiated to evaluate the response of plants and trees to pre-commercial thinning treatments [41]. The TWYGS comprise a randomized complete block design with replicates of four experimental treatments distributed across the TNF and established from 2002 to 2004. Manipulated sites on POW consisted of an un-thinned control (young-growth) stand paired with two thinned stands [41]. In 2010, we selected six study sites across northern Prince of Wales Island (POW), Alaska (55.9° N, 132.9° W; Fig 1); the largest island in the Alexander Archipelago at 6,674 km^2^. Our sites included seven TWYGS treatments, six un-thinned young-growth stands, three old-growth stands and three clearcuts (for a total of 19 stands). In 2011 and 2012 we added one old-growth and one clearcut grids (21 grids total). This sampling design encompassed a wide range of habitat conditions and successional stages across POW. Pre-commercially thinned stands had a robust understory with remaining trees spaced according to thinning treatment (e.g. 14 × 14 or 18 × 18 ft). Young-growth stands had little spatial variation and were composed of densely regenerated, even-aged, small-diameter trees with sparse understory. Old-growth stands ranged from low-elevation, high-productivity to high-elevation, mesic sites. A variety of tree size classes and ages contributed to a more heterogeneous canopy in this stand type [42]. Clearcuts ranged in age from 1–15 years post-harvest, contained few remaining overstory trees, and were characterized by a dense layer of residual timber slash mixed with regenerating shrubs and tree seedlings.

Annual precipitation on POW ranges from 254–508 cm and average temperatures range from 10–17°C in summer to 0–6°C in winter. Elevation at our study sites varied from 0 to 305 m. Areas lower than ~600 m are characterized by temperate, coniferous rainforest [43], dominated by Sitka spruce (*Picea sitchensis*) and western hemlock (*Tsuga heterophylla*). The understory consists primarily of *Vaccinium* spp., false azalea (*Menziesia ferruginea*), and salmonberry (*Rubus spectabilis*). The herbaceous layer includes skunk cabbage (*Lysichiton americanum*) and bunchberry (*Cornus canadensis*), among various other forbs, mosses, and ferns. Muskegs, or peatland bogs, compose the majority of natural non-forested areas on POW.

Similar to other high latitude islands, POW is characterized by a depauperate fauna with mink (*Neovison vison*) and ermine (*Mustela erminea celenda*) the only native mesocarnivores and few small mammals including Keen’s mice, long-tailed voles (*Microtus longicaudas*), dusky shrews, and northern flying squirrels (*Glaucomys sabrinus griseifrons*) [32]. Long-tailed vole densities, which were high in the 1970s [33], are currently so low that in multiple years of attempts by several different research teams, only a handful have been captured ([44,45], S. MacDonald, pers. comm, this study). American marten (*Martes americana*) were introduced to POW in the 1930s to provide trapping opportunities for rural residents. The avian fauna of the island is also limited, with dark-eyed junco (*Junco heymalis*) the only ground-nesting songbird. Northern goshawk (*Accipiter gentilis*), a raptor that rarely preys on small mammals, is the main avian predator.

### Small mammal trapping

We live-trapped small mammals from May to August 2010–2012 using 40 to 52 Sherman live-traps (H.B. Sherman Traps, Inc, Tallahassee, FL, USA) per grid set at 25-m intervals and configured to fit within each forest stand. We baited traps with a mix of oats, molasses and peanut butter. Polyester bedding was provided to aid in thermoregulation. Each year, trapping occurred for five consecutive nights three times in late spring, early summer and late summer/fall following a robust-design capture-recapture protocol [46]. We checked traps once in the morning. Each trapped individual was weighed, sexed, aged, and assessed for reproductive status. Mice were marked with a passive integrated transponder (PIT) tag (Biomark, Boise, ID, USA) for permanent identification after a brief exposure to Isoflurane (Piramal Healthcare Limited, Andhra Pradesh, India). After immobilization, we collected blood via tail clipping [47] from all mice at first capture. Live-captured shrews were released without processing. Carcasses of all incidental mortalities (255 mice, 1330 shrews) were deposited in the Vertebrate Museum at the University of Wyoming (UW) Berry Center for Biodiversity Conservation. All procedures were approved by the Institutional Animal Care and Use Committee at UW (see S1 Text) and adhere to the guidelines of the American Society of Mammalogists [48]. Our trapping permit was obtained from the Alaska Department of Fish and Game and study methods were approved by the US Forest Service, Tongass National Forest, Thorne Bay District. This study did not involve endangered or protected species.

### Population modeling

We generated estimates of survival and abundance for Keen’s mice from mark-recapture data using the robust-design population model in Program MARK [49]. We constructed several *a priori* competing models (Table A in S1 Text) and selected among them based on Akaike’s information criterion values corrected for sample size (AICc), and the significance of parameter estimates. Models were developed separately for each grid for the three years and 45 occasions (three primary and five secondary occasions in each sampling year). We used unequal time intervals (1 month between the summer primary sessions and 10 months for the overwinter periods) to generate survival estimates.

For dusky shrews we used a dead-and-alive framework to generate abundance estimates. After removing the trap mortalities from the dataset for each grid, we calculated the abundance of the live animals with the Horvitz-Thompson estimator [46]. Because we had no marked individuals, we used estimates of capture probability (and 95% confidence intervals) for *Sorex* shrews from a study by Otto and Roloff [50]. We treated the number of live captures as minimum number known alive (MNKA). We then added the number of trap mortalities to the live estimates to generate the total abundance estimates. To assess the potential effect of trap mortality on the shrew population, we calculated growth rate (lambda) from abundance estimates by dividing values in sessions 2 and 3 by sessions 1 and 2 in each year. We then regressed the resulting lambda values against trap mortality in the preceding session. Data for additional population indices (MNKA and captures per 100 trap nights [100TN]), as well as the relationships among them for mice (S1 Fig) and shrews (S2 Fig) are provided in the supplementary materials.

We used a traditional boundary-strip method to estimate mouse densities. We calculated the mean maximum distance moved (MMDM; average 52 m) by measuring the distances traveled between subsequent captures [51]. We calculated the effective trapping area (ETA) by adding a 50 m buffer to the area covered by live-traps. We then divided the abundance estimate by the ETA for each trapping session per grid. Shrew densities were calculated by dividing the estimated abundance by the effective trapping area calculated for each grid based on mice MMDM. On a subset of grids we used spatially explicit capture-recapture functions [52] to estimate density of mice with the package secr in the R statistical environment ([53,54]; S1 Text). Similar to observations by Gerber and Parmenter [55], our models yielded higher estimates compared to MMDM (S3 Fig). Given these results and our interest in vital rates we did not further pursue estimates using the Efford and Fewster [52] methods.

### Habitat measurements

We conducted vegetation sampling three times each summer at eight randomly selected trapping stations in each grid in 2010 and twice each summer at nine trapping stations in 2011 and 2012. For understory components, two 20-m transects were placed in the four cardinal directions, crossing at the center of the trap station. We used line-intercept methods to record percent cover of shrub species, coarse woody debris, and herbaceous plants along transects in all years. In 2010, at the end of each transect, we sampled a 1 × 1 m plot to obtain occurrence for shrubs and herbaceous species. In all years, we collected and weighed all epigeous fungi, truffles, and earthworms, and counted the number of conifer cones in the same plots. In 2010, pitfall traps were used to sample soil macro-invertebrates (mostly beetles) every 5 m along one randomly selected transect. All invertebrates were later identified to family. To characterize the overstory, we measured leaf-area index (LAI) at each sampled trap station in 2010 [42]. Averages of understory and overstory variables represent stand-level values for each trapping grid. We obtained the elevation at each grid from a digital elevation model using ArcGIS software [56]. Each grid was characterized by 11 variables (Table B in S1 Text). We reduced the dimensionality of the habitat variables by non-metric multidimensional scaling (NMDS; [57]) in Program R. We used variable scores (*r* > 0.40) to interpret the dimensions. Loading of these variables on NMDS1 and NMDS2 are provided in Table C in S1 Text.

### Competition analysis

We used densities of mice and shrews and habitat variables to estimate competition coefficients (α). We first standardized density estimates for each species to a mean of zero and a standard deviation of one [38]. This eliminated any dependence of α on the density variances [58]. We included NMDS scores to represent habitat variables. We also included elevation as a separate predictor variable in candidate models because of high variation. The effect of Keen’s mouse density and habitat variables on dusky shrew density can be estimated by:

$$Y_{s}=a+b_{p}X_{p}+b_{1}X_{1}+\cdots +b_{n}X_{n}$$ (1)

where Y_s_ is the density of shrews, b_1_…b_n_ and X_1_…X_n_ are the coefficients and associated predictor variables, respectively, X_p_ is the density of mice, and b_p_ is the α coefficient, estimating the effect of mice on shrews. In these analyses we used density estimates for both species of small mammals from each session and overstory variables from 2010 (assuming overstory cover changed little over the course of the study) and understory variables derived for each grid each summer.

Because animal abundance is a function of the net of reproduction and mortality, we also constructed models with the density of shrews in the previous session as one of the potential explanatory variables [i.e., auto-regressive models; 59]:

$$Y_{s}=a+b_{s(t-1)}X_{s(t-1)}+b_{p}X_{p}+b_{1}X_{1}+\cdots +b_{n}X_{n}$$ (2)

where b_s(t-1)_ is the regression coefficient and X_s(t-1)_ is the density of shrews in the previous session. We only included density estimates from sessions 2 and 3 of each year as the dependent variables and session 1 and 2 as the independent ones.

Because mouse density varied by year, we performed separate regressions for 2010, 2011, and 2012. We used generalized linear models (glm) in R to construct models and compare among them using AICc, *R*^*2*^, and parsimony. We created 15 regressive (Table D in S1 Text) and 16 auto-regressive models containing all configurations of NMDS dimensions, elevation, and densities of each species.

### Stable isotope analysis

To assess dietary overlap of shrews and mice, we used stable isotopes of carbon (δ^13^C) and nitrogen (δ^15^N). Other methods were not applicable because stomachs and feces of both species largely contained bait. We analyzed whole blood collected from mice and muscle tissue from mice and shrew mortalities. We sampled diet items including macro-invertebrates (beetles and earthworms), conifer seeds, berries, lichen, and fungi from all grids. We dried whole blood (mice), muscle (mice and shrews), and potential food samples at 60°C for 48 h and then homogenized them in a ball mill (Mixer Mill MM200, Retsch Inc., Newtown, PA, USA). Samples were weighed in duplicate and sent to the University of Wyoming Stable Isotope Facility. Analyses of δ^13^C and δ^15^N were conducted with a Costech 410 elemental analyzer (Costech Analytical, Valencia, CA, USA) connected to a Thermo Finnigan Delta^PLUS^ XP Continuous Flow Isotope Ratio Mass Spectrometer (Thermo Fisher Scientific, Inc., Waltham, MA, USA). Results, reported as parts per thousand (‰) in relation to internal laboratory standards, were accepted (and averaged) when the variance between duplicate samples was lower than that of the standards within each run.

We compared the isotope values of whole blood and muscle tissue from mice using a K-nearest neighbor randomization test [60] in R to ensure they were equivalent. We also tested for differences among the diet items and compared the δ^13^C and δ^15^N of mice and shrews by habitat type (young growth, thinned, old growth and clearcut) each year using the KNN test. The isotope data were converted to estimates of relative contributions of dietary items separately for mice and shrews in each habitat type using the multiple source, dual-isotope linear mixing-model package SISUS in R [61]. Because the variation in shrew signatures was low we used population means per grid for shrews. In contrast, we calculated dietary contribution for individual mice because of the large variation associated with those data. We corrected source data for consumer-diet discrimination by 1‰ for δ^13^C and 3‰ for δ^15^N for mice [62,63] and 1.3‰ for δ^13^C and 2.5‰ for δ^15^N for shrews [64]. We implemented concentration-dependent models to account for differences in C:N of sources. We then calculated the overlap in diet between each mouse and the population of shrews in each grid and considered it significant when both species included a contribution > 20% of a diet item.

To describe the isotopic niches of the two species we used kernel utilization density methods (KUD; [65]). We extracted the 50%, 75% and 95% contours with the rKIN package in R [66] from the probability surface (S4 Fig). We then calculated niche size and percent overlap for mice and shrews with rKIN in 2010–2011 and 2012. Because the number of samples available in 2012 was low (21 mice and 64 shrews; as a result of fewer caught mice and lower trap mortality in shrews) we randomly subsampled the 2010–2011 data (280 mice and 231 shrews) with the number of points in 2012. We calculated niche size and percent overlap for this subsample and compared it to the original, full dataset.

### Whole community energy consumption

Theoretically, when released from competition, the weaker competitor should increase in numbers such that the change in abundance is proportional to the inverse of average metabolic rate [40]. Because the metabolic rate of shrews is higher than expected based on allometric expectations (i.e., ¾ of body mass; [67]), it is likely that their numerical increase will be lower than predicted by body mass alone. To assess the effects of declines in Keen’s mice, the dominant competitor, on dusky shrews we estimated the whole community energy consumption by multiplying the abundance of each species by the average metabolic rate of individuals in each year of study. We calculated this value for the last trapping session in each year (i.e., August) when the abundance of both species reached a maximum. For shrews we used values for field metabolic rate (FMR; Kj per day) published by Ochocińska and Taylor [68] for common shrews (*Sorex araneus*). Because common shrews are slightly larger (8.2g) than dusky shrews we converted their estimates to mass specific FMR and used the average body mass of shrews in each year to generate values for individuals. For mice we used FMR reported by Degen [69] for *Peromyscus maniculatus*, which is closely related to *P*. *keeni* [70]. The average body mass of Keen’s mice in 2010, 2011, and 2012 was 21.2g, 20.3g, and 20.9g, respectively. We then calculated the total energy consumption of the community by summing the values of mice and shrews in each year.

## Results

### Abundance and density

**Fig 2:**
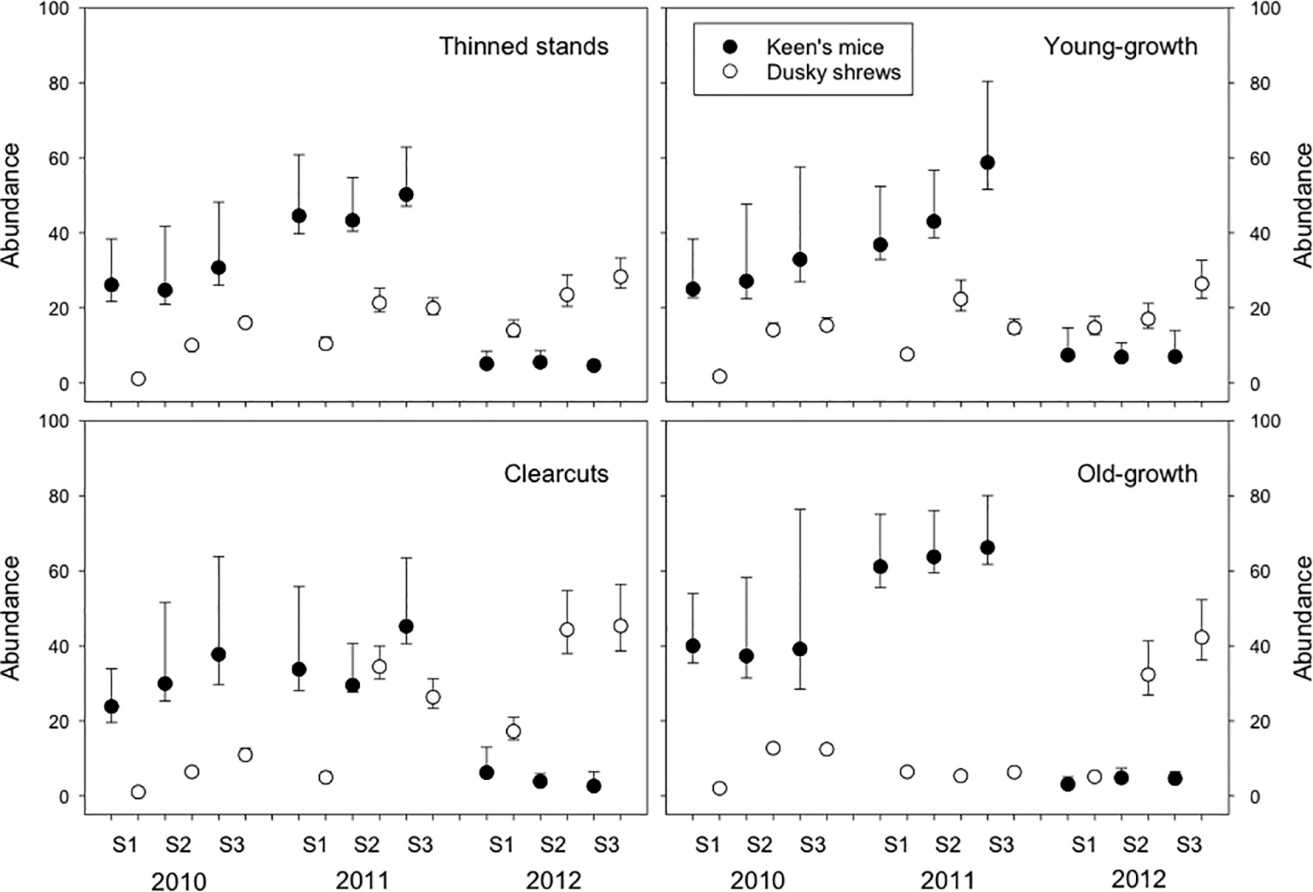
Abundance estimates (± 95% confidence intervals) for Keen’s mice and dusky shrews. Abundance was estimated in each of four habitat types on Prince of Wales Island, Alaska. Every grid was trapped during three sessions (S1-S3) each summer from 2010–2012.

We captured a total of 2,543 individual Keen’s mice and 1,744 dusky shrews in all habitats and study sites in all years. In contrast, we captured only 18 long-tailed voles and 36 northern flying squirrels. There was strong correlation between abundance estimates and indices of population size for both species (supplementary materials S1 and S2 Figs). Abundance estimates show that mouse populations in our study area peaked in 2011 and crashed in 2012 (Fig 2).

**Fig 3:**
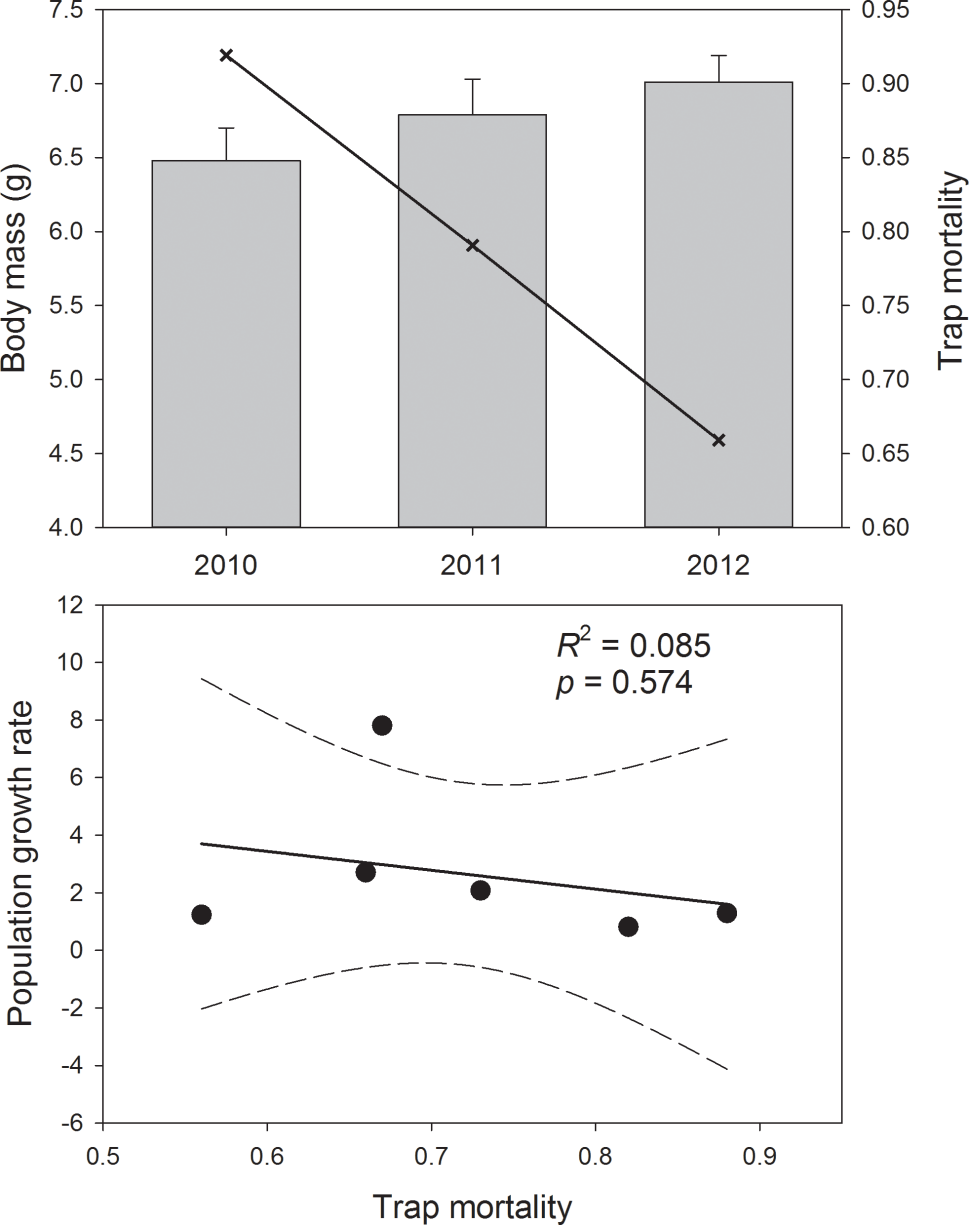
Body mass, mortality of trapped shrews, and relationship to population growth rate. Average body mass (bars; ± 95% confidence intervals), trap mortality rate (line; top), and the relation between trap mortality and population growth rate (bottom) of dusky shrews captured in 2010–2012 on Prince of Wales Island, Alaska. Letters indicate significant differences.

**Fig 4:**
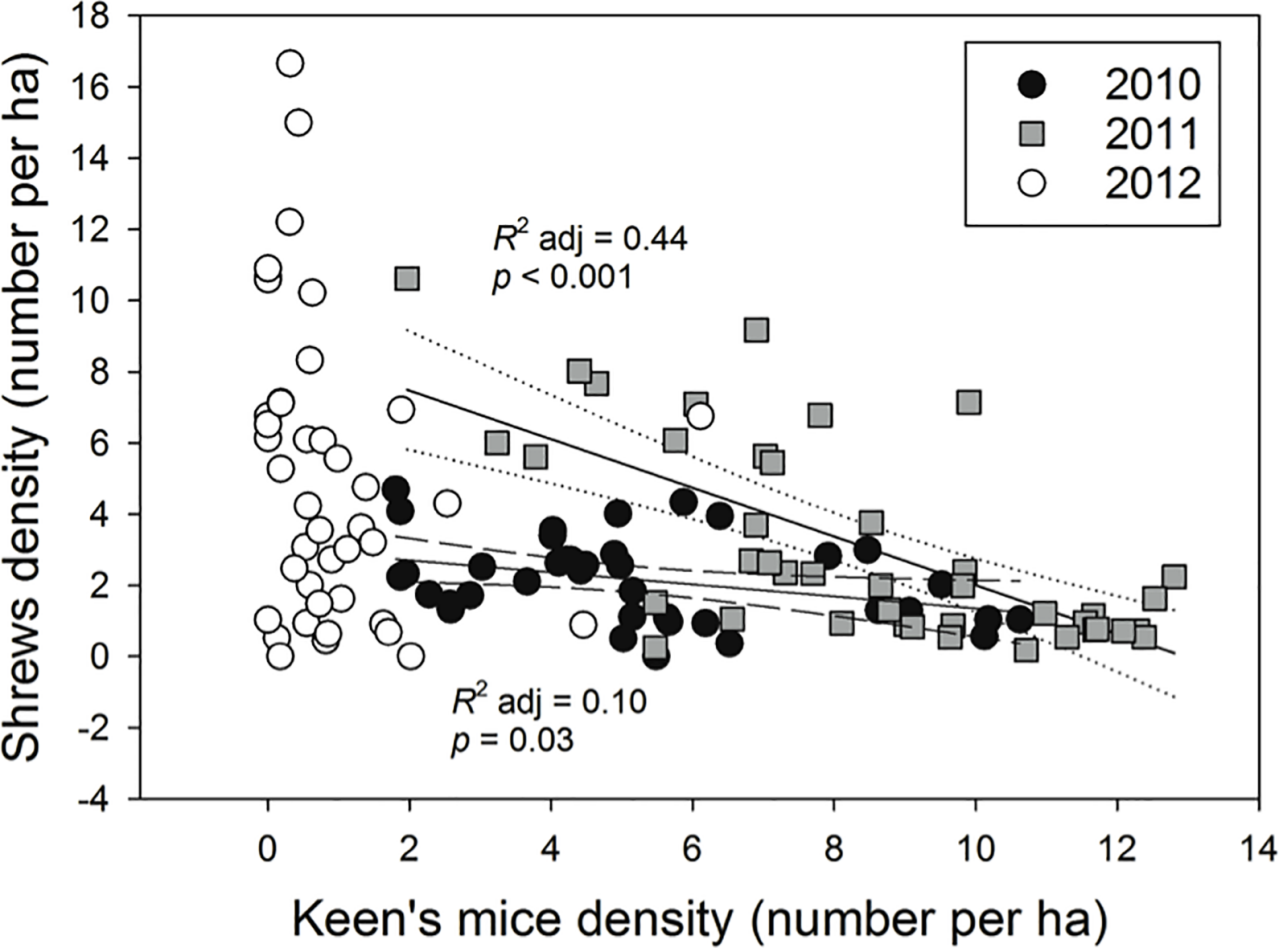
Relationship of shrew and mouse densities. Density of dusky shrews on Prince of Wales Island, Alaska from 2010–2012 in relation to the density of Keen’s mice.

Concomitantly, high overwinter mortality of dusky shrews was followed by steady increases during the breeding season in all years, reaching the highest abundance in 2012 (Fig 2). This increase was pronounced in all habitat types except thinned stands. Shrew body mass was significantly lower and trap mortality higher in 2010 than in 2012 (Fig 3). Nonetheless, trap mortality had little effect on population growth rate of shrews (Fig 3). In contrast, mortality of mice in traps was low (2010: 13.5%, 2011: 6.5%, 2012: 13.0%) and unrelated to body mass or population growth. There was a significant inverse relationship of Keen’s mice and dusky shrew densities in 2010 and 2011 (*p* = 0.03 and *p* < 0.001, respectively; Fig 4), with low shrew densities in areas characterized by high mice densities (Fig 4). There was no relation between mice and shrew densities in 2012 (*p* = 0.16).

### Competition models

**Fig 5:**
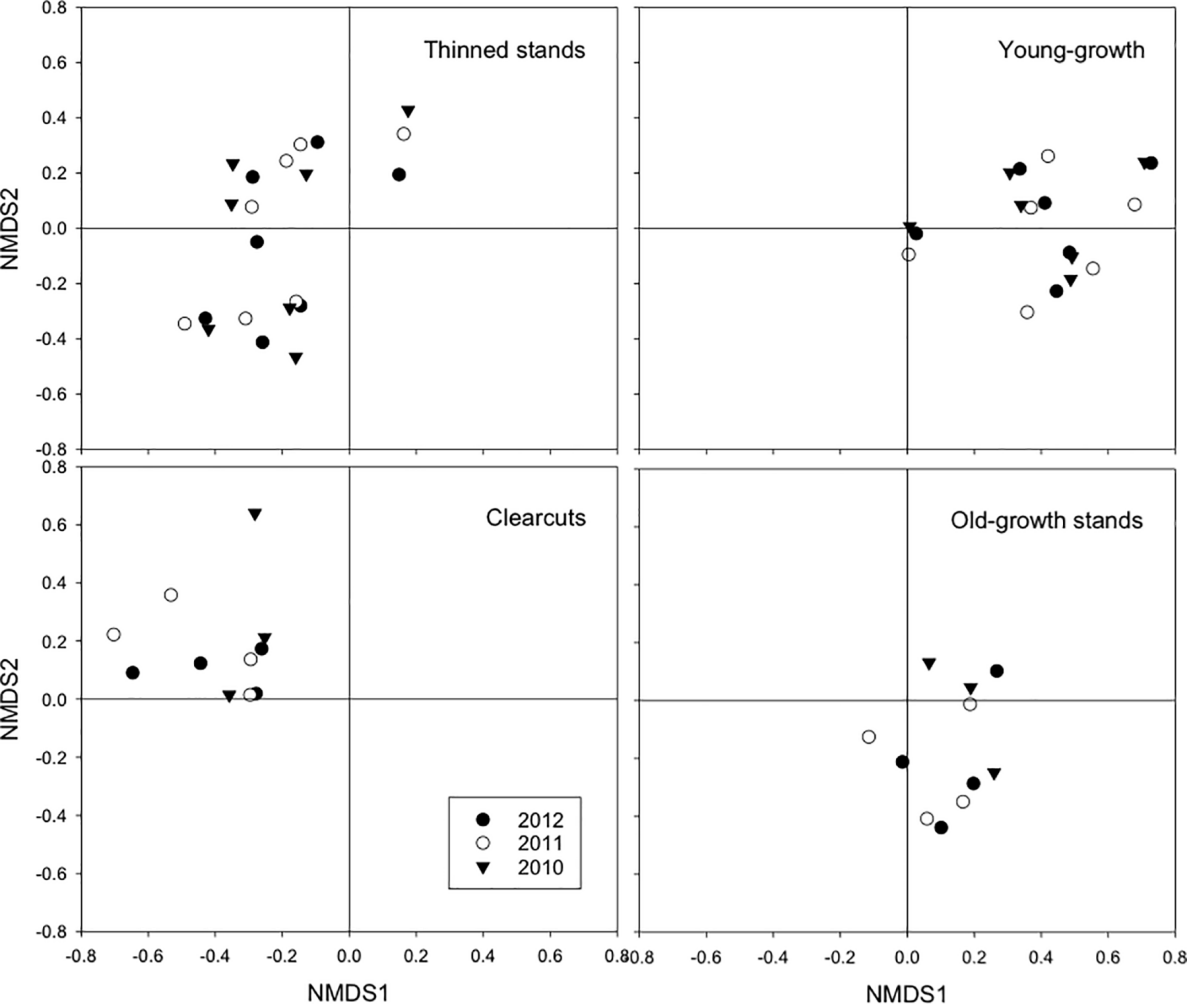
Values of non-metric multidimensional scaling (NMDS). NMDS1 and NMDS2 values for habitat and food variables in each of four habitat types on Prince of Wales Island, Alaska from 2010–2012.

Habitat variables were reduced to two dimensions (Table C in S1 Text) representing overstory and understory cover, and species diversity, respectively. These two dimensions separated the four habitat types with most of the divergence occurring on NMDS1 and some on NMDS2, especially between old growth and clearcuts (Fig 5).

**Fig 6:**
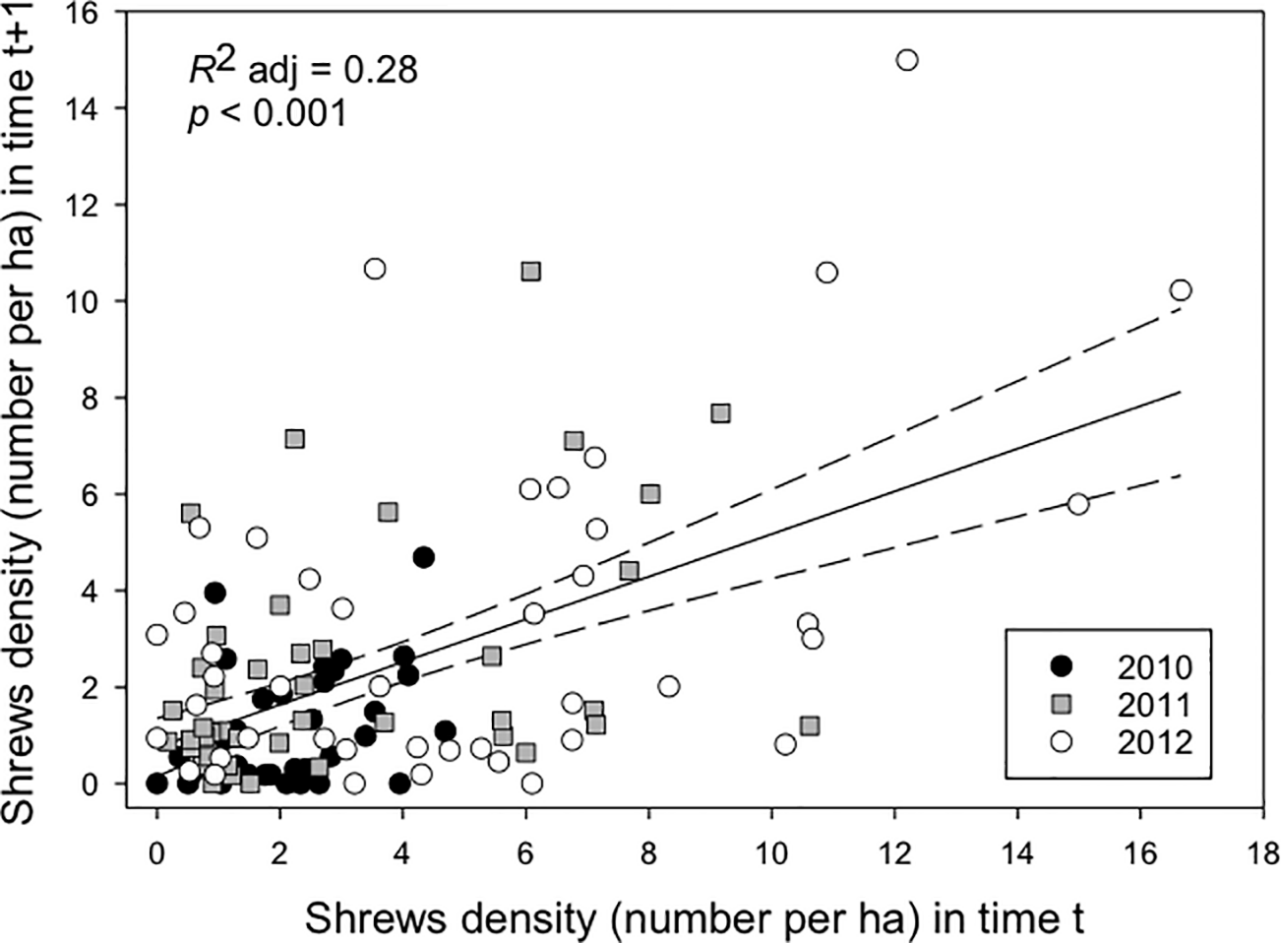
Relationship between shrew density in the current session vs the previous one. Density of dusky shrews in the current trapping session (2 or 3) on Prince of Wales Island, Alaska, in relation to their density in the previous session (1 or 2) in each year from 2010–2012. The relationship is shown for all years combined.

There was a positive relationship between shrew densities in time *t* (sessions 2 or 3) and those in time *t*-1 (sessions 1 or 2; *p* < 0.001; Fig 6). Additionally, the auto-regressive models exhibited higher *R*^2^ values than the regressive ones (Table 1 and Table D in S1 Text), suggesting that models excluding information on shrews did not sufficiently explain the variation in their densities.

**Table 1:** Generalized linear auto-regressive models of dusky shrew density and estimated competition coefficients (α) on Prince of Wales Island, Alaska.

Year	Model	AICc	ΔAIC	*R*^*2*^	*α*
2010	**SOMO**_**t**_ **= SOMO**_**(t-1)**_ **+ PEKE**	**34.6**	**0.00**	**0.18**	**-0.14**
	SOMO_t_ = SOMO_(t-1)_	34.7	0.01	0.13	-
	SOMO_t_ = SOMO_(t-1)_ + Elevation	36.0	1.35	0.15	-
	SOMO_t_ = SOMO_(t-1)_ + PEKE + NMDS1	36.4	1.73	0.20	-0.16
2011	**SOMO**_**t**_ **= SOMO**_**(t-1)**_ **+ PEKE**	**87.2**	**0.00**	**0.61**	**-0.90**
	SOMO_t_ = SOMO_(t-1)_ + PEKE + Elevation	88.7	1.45	0.62	-0.89
	SOMO_t_ = SOMO_(t-1)_ + PEKE + NMDS1	89.0	1.72	0.62	-0.96
2012	SOMO_t_ = SOMO_(t-1)_ + PEKE + Elevation	135.5	0.00	0.44	-1.15
	SOMO_t_ = SOMO_(t-1)_ + NMDS2 + Elevation	136.3	0.79	0.43	-
	**SOMO**_**t**_ **= SOMO**_**(t-1)**_ **+ Elevation**	**136.4**	**0.95**	**0.39**	**-**
	SOMO_t_ = SOMO_(t-1)_ + PEKE + NMDS2 + Elevation	137.0	1.51	0.45	-0.90
	SOMO_t_ = SOMO_(t-1)_ + NMDS1 + Elevation	137.1	1.58	0.41	-

For all years, the dependent variable was shrew density (SOMO) at time *t*. Independent variables were shrew density at time *t*-1 (trapping sessions 1 or 2), Keen’s mice density (PEKE) at time *t* (trapping sessions 2 or 3), elevation at the trapping grid, NMDS1 and NMDS2. Only models with considerable support (ΔAIC ≤ 2) are shown. In bold are top models based on AICc, *R*^2^ and parsimony (also see Fig 4).

In 2010 and 2011, the top auto-regressive models only included the density of mice as an added predictor (Table 1). The associated α coefficients indicate a large increase in the influence of mice on shrews in 2011 (α = -0.14 and -0.90, respectively). In 2012, the best fitting model only included shrew density in *t*-1 and elevation, with densities increasing with altitude (Table 1).

### Diet composition and niche overlap

**Fig 7:**
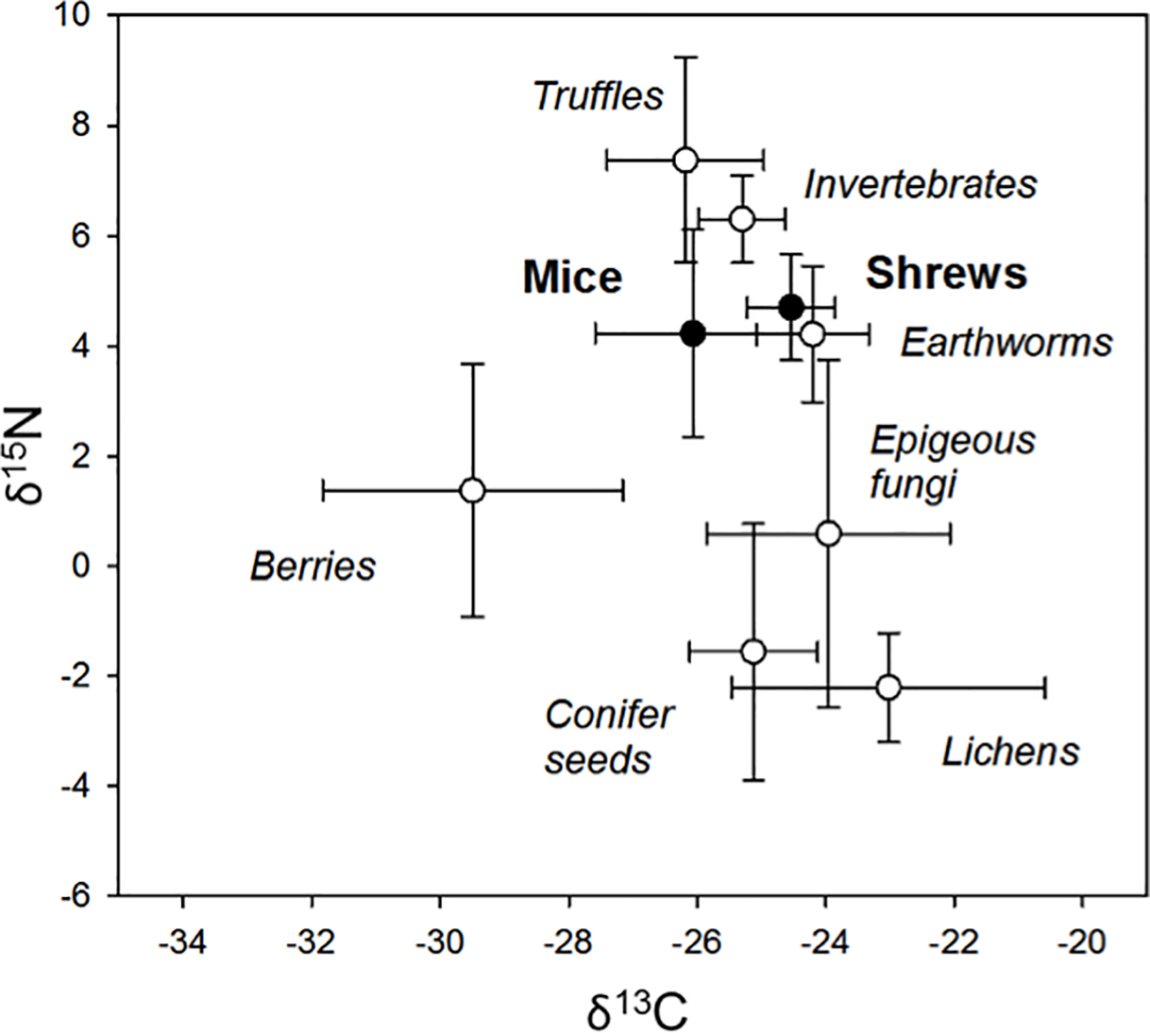
Stable carbon and nitrogen values for Keen’s mice, dusky shrews and the potential foods. Values of δ^13^C and δ^15^N (average ± 95% confidence intervals) for blood and muscle tissues of Keen’s mice and dusky shrews captured on Prince of Wales Island, Alaska 2010–2012. Potential diet items were corrected for consumer-diet discrimination by using a trophic enrichment factor of 1.3‰ for δ^13^C and 2.5‰ for δ^15^N in relation to shrew diets.

We found no significant differences in the δ^13^C and δ^15^N of whole blood and muscle tissue from mice (*p* > 0.05) and combined these samples. All diet items in each habitat type exhibited significant differences in δ^13^C and δ^15^N (*p* < 0.05). Macro-invertebrates were pooled into a single group with the exception of earthworms, which were isotopically distinct (Fig 7).

The variance in diets of mice was double that observed in shrews (Fig 7). Earthworms and other macro-invertebrates had the largest contributions to the diets of these small mammals (29–58% and 60–71%, respectively; Table 2). Additionally, in young-growth stands, mushrooms substantially contributed (34%) to the assimilated diet of dusky shrews. The proportion of mice that included > 20% of invertebrates, mushrooms, or earthworms in their diet varied, with the highest overlap with shrew diets occurring in young-growth and old-growth stands (Table 2).

**Table 2:** Proportion of food items in the diet of dusky shrews (mean ± SD) and percent of individual Keen’s mice that include > 20% of specific prey sources in their diet in each habitat type on Prince of Wales Island, Alaska, in 2010–2012.

	Proportion in the diet of shrews			
Food Item	Clearcut (42)	Young Growth (61)	Thinned (85)	Old Growth (43)
Invertebrates	**0.29 (± 0.01)**	**0.58 (± 0.01)**	0.08 (± 0.05)	**0.29 (± 0.01)**
Mushrooms	0.03 (± 0.03)	**0.34 (± 0.03)**	0.19 (± 0.06)	0.05 (± 0.04)
Earthworms	**0.66 (± 0.03)**	0.06 (± 0.05)	**0.71 (± 0.11)**	**0.60 (± 0.04)**
	Percent of individual mice			
Food Item	(27)	(65)	(71)	(39)
Invertebrates	19	12	-	44
Mushrooms	-	43	-	-
Earthworms	11	-	31	-

Proportion in the diet and percent of individuals was estimated from δ^13^C and δ^15^N of shrews and mice and their potential prey using a dual-isotope linear mixing model. Diet items were corrected using a discrimination factor of 1.3‰ for δ^13^C and 2.5‰ for δ^15^N for shrews and 1‰ for δ^13^C and 3‰ for δ^15^N for mice. Sample sizes are provided in parentheses. Bolded food items constituted > 20% of the assimilated diet of shrews.

**Fig 8:**
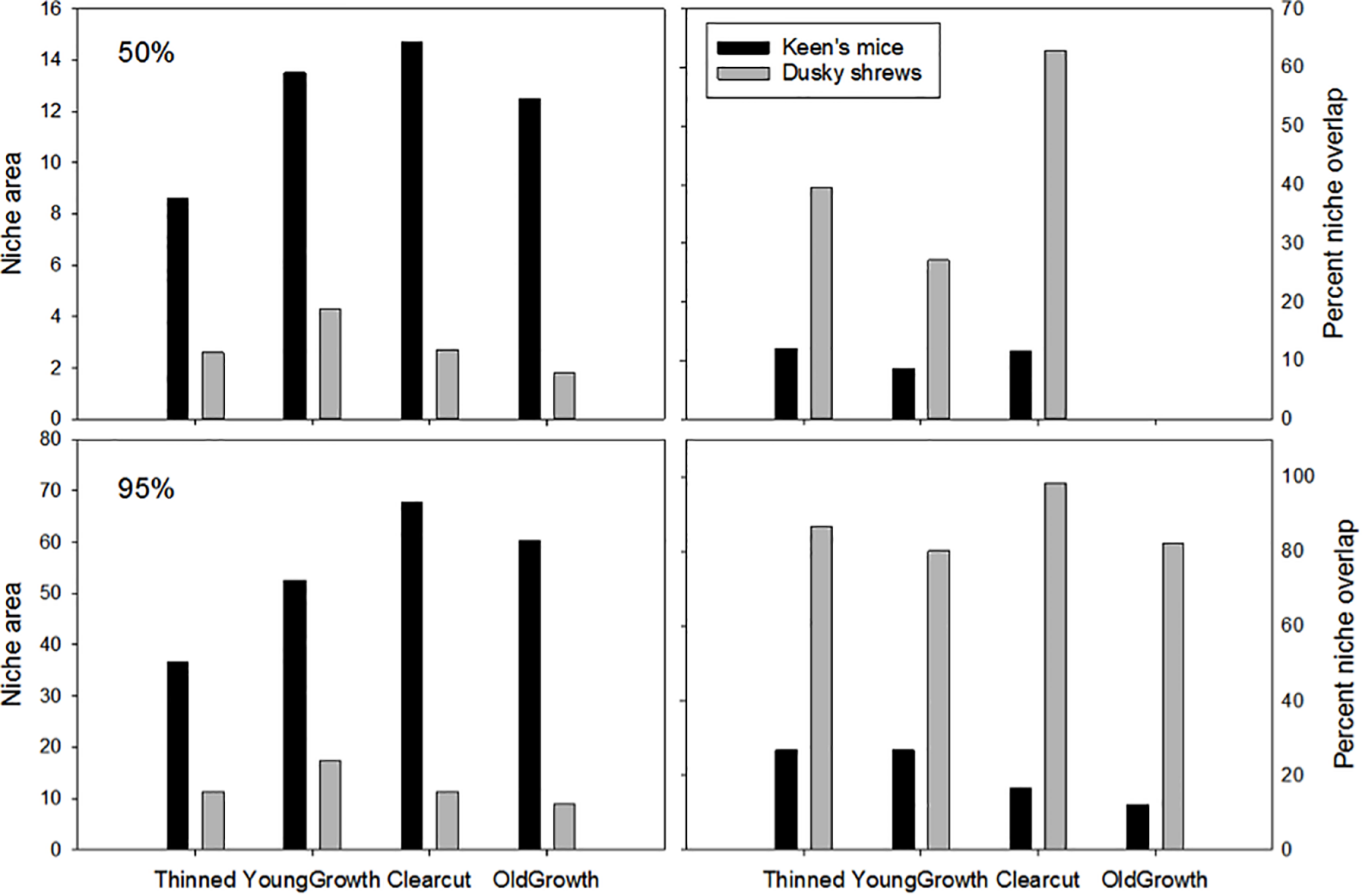
Isotopic niche size and percent overlap. Niche size and percent overlap of Keen’s mice and dusky shrews on Prince of Wales Island, Alaska 2010–2011 (bottom) from the 50% and 95% kernel utilization density contours (see also supplementary materials S4 Fig) in each of four habitat types.

Mice niche (KUD) was nearly 5× larger within the 95% contour than within the 50% contour, whereas for shrews the 95% niche was only twice that of the 50% (Table 3), indicating that their diets were more uniform. Subsampling of the 2010–2011 dataset with the number of data point available in 2012 demonstrated that the difference between the two periods was largely a result of contraction of the dietary niche in mice and a shift in the niche of shrews rather than an artifact of sample size (Table 3). At the 50% level, the niche size of mice overlapped largely with that of shrews expect in old-growth stands; at 95% shrew niches were nearly completely encompassed by mice in all habitats (Fig 8). Between 2010–2011 and 2012, niche size of mice decreased by 32–33% in all contours (Table 3). Concurrently the percent overlap between the two species increased from 13% of the dusky shrews 95% contour to a considerable 53% in the core 50% (Table 3). Dusky shrew core niche (50%) in 2012 only overlapped with that of 2010–2011 by 53.4%, suggesting a large shift in resource use between these two periods. This was also noticeable in the 75% (64.5% overlap) and 95% contour (69.1% overlap).

**Table 3:** Isotopic niche size and percent overlap of Keen’s mice and dusky shrews on Prince of Wales Island, Alaska, in 2010–2011 and 2012, based on 50%, 75% and 95% kernel utilization density (KUD) contours.

Contour	2010–2011			
	Niche size		Percent overlap	
	Keen's mice	Dusky shrews	Keen's mice	Dusky shrews
50	10.9	2.8	8.7	33.7
75	22.5	5.8	16.1	62.2
95	48.0	13.5	21.1	75.4
	2010–2011 sub-sampled			
50	9.3 (3.2)	2.7 (0.7)	12.2 (11.8)	35.1 (26.2)
75	18.4 (5.7)	5.6 (1.2)	17.6 (12.7)	57.2 (21.9)
95	37.0 (10.6)	12.3 (2.3)	24.6 (10.8)	70.8 (17.9)
	2012			
50	7.5	2.9	28.0	72.0
75	15.0	6.0	31.6	78.4
95	32.1	11.6	31.2	86.5

Data from 2010–2011 were randomly subsampled 5 times for the sample size of 2012. Confidence intervals (95%) are reported in parentheses.

### Whole community energy consumption

**Fig 9:**
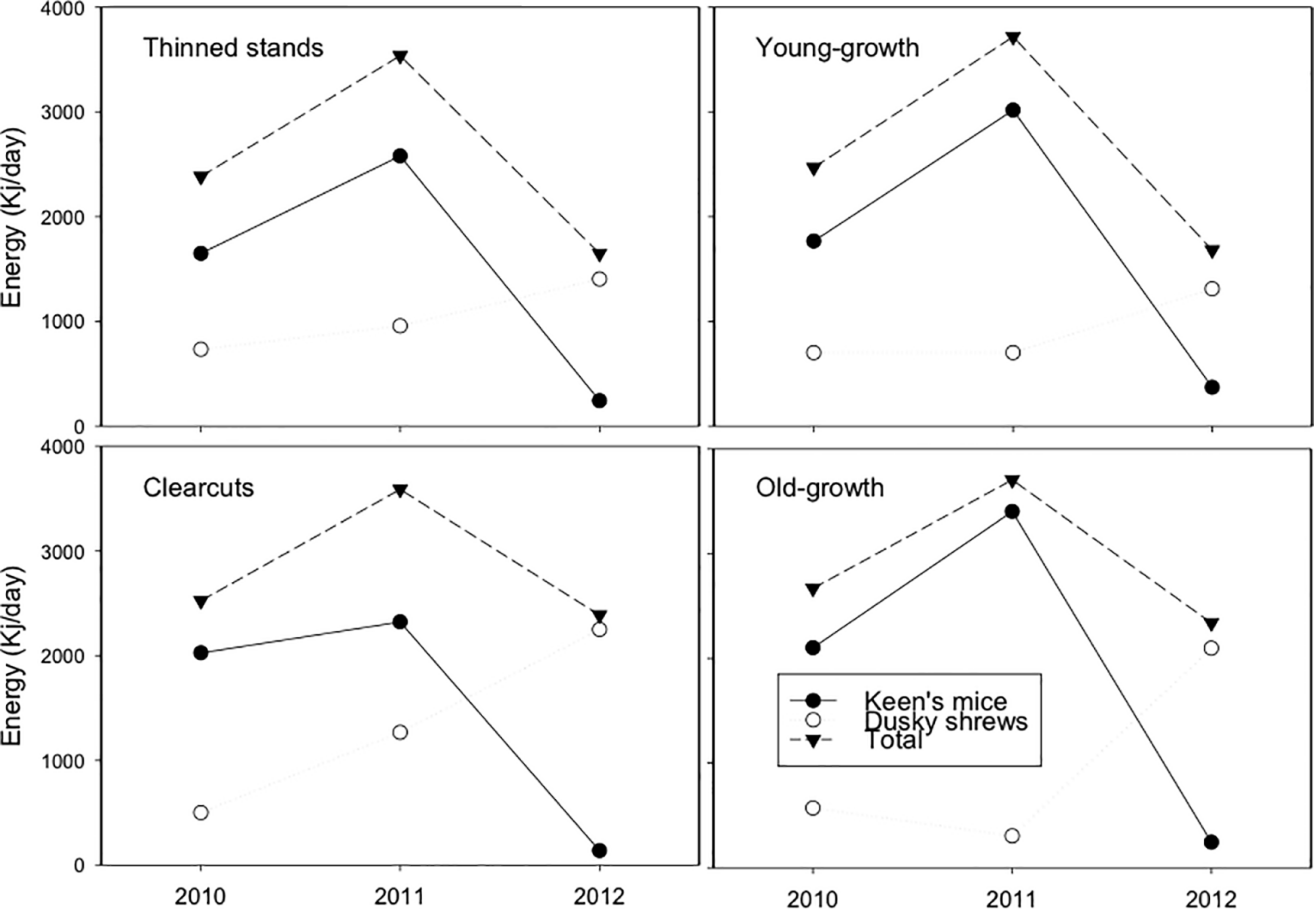
Total energy consumption of mice and shrews (Kj/day). Energy consumption was calculated based on abundance and species-specific field metabolic rates in four habitat types on Prince of Wales Island, Alaska 2010–2012. The increase in shrews partially compensated for the decline in mice in 2012.

The total energy consumption of Keen’s mice and dusky shrews increased between 2010 and 2011 (Fig 9) mirroring the increase in mice abundance in all habitats (Fig 2). It then declined in 2012 and reached approximately 69 and 68% of the 2010 and 47 and 45% of the 2011 values in thinned and young-growth stands, respectively. In old growth, total energy consumption reached 88% of 2010 and 63% of 2011 levels, whereas in clearcuts these values were 95% and 67%, respectively. This suggests that in these two habitats, shrews compensated for the declines in mice abundance more significantly than in thinned and young-growth stands.

## Discussion

Our results provide several lines of evidence that interspecific competition influences the densities of mice and shrews in Southeast Alaska, but that the effects of competition are spatially and temporally dynamic. Similar to removal experiments, we observed the expected increase in densities of dusky shrews after mouse populations crashed in 2012. Although shrew abundance in 2012 increased above 2010–2011 levels in all habitat types, this was most pronounced in clearcut and old-growth stands. Indeed, in these two habitats the increase in shrews partially compensated energetically for the declines in mice. In addition, α coefficients revealed that the influence of Keen’s mice, the dominant competitor, varied congruent with their densities. The numerical responses of shrews occurred in conjunction with habitat-specific segregation in diet. When mouse populations were low, niche overlap between mice and shrews increased as the diet of shrews shifted, indicating that in the absence of their competitor, shrews were able to exploit previously unavailable resources. Our results clearly illustrate that competition can occur even among species that differ in size and diet and that the evolutionary adaptations of one competitor may limit the ability of the community to compensate energetically. These complex interactions, observed in a relatively simple ecological system, highlight the difficulty in assessing the role of biotic factors in structuring communities and may explain the recent finding of strong abiotic masking of competition and negative covariances [9].

In 2010 estimated α coefficients suggested weak competitive interactions concurrent with high dietary overlap between mice and shrews. Intraspecific competition for food regulates small mammal populations in other systems [71]. Although we were unable to measure food limitation per se, our habitat variables included measures of food availability. Biomass of earthworms and numbers of beetles exhibited low scores on the two NMDS axes, both of which explained little of the variation in shrew densities. In addition, in all years, there was a positive relationship between shrew density in the current session and that in the following one, negating expectations of intraspecific competition. Nonetheless, in 2010, shrew body condition was low. It is possible that competition with mice for specific foods that enhance body condition in shrews and that were less available in 2010 resulted in competitive effects at the individual, rather than the population level. Alternatively, other independent processes such as parasite load or disease, [72,73] may have influenced shrew populations in 2010.

Although habitat variables did not exert a strong influence on competition, the four habitat types differed greatly in canopy and horizontal cover (NMDS1) and food availability (NMDS2). In addition, niche overlap and the numerical responses of shrews to competition with mice differed among habitats. In old-growth stands in 2010–2011, there was little overlap in diets of mice and shrews. Concurrently, in these grids shrew density was low until mice numbers crashed, after which they substantially increased. In contrast, in clearcuts, the isotopic niche of shrews was nearly fully encompassed by that of mice while their numerical responses were similar to old growth. This type of habitat-dependent competition has also been shown in Australian rodents where *Rattus lutreolus* and *Pseudomys gracilicaudatus* coexist in both wetter and drier habitats [26]. In drier sites, they displayed symmetric competition but within wetter sites, *Rattus* reduced the density of *Pseudomys* with no mutual effect.

Despite the vast differences in habitat structure (NMDS1) and food availability (NMDS2) between thinned and young-growth stands, the numerical response of shrews to the crash of mice was dampened and the mice isotopic niche overlapped less with that of shrews, especially in thinned stands. In these habitats, the increase in shrew abundance was insufficient to compensate energetically for the decline in mice, especially compared with 2011. It is possible that in young-growth stands shrews had low food availability even in the absence of mice, which limited their potential increase. In contrast, thinned stands encompass much of the diversity, and thus food availability, observed in the other three habitat types. Nonetheless, although we and other studies [36] have shown that dusky shrews consume mushrooms and lichens, as insectivores they are unlikely to take advantage of all food types consumed by mice (e.g., berries). This may also explain our observation that shrews only partially compensated energetically for the decline in mice even in old-growth stands and clearcuts. Others have suggested that limitations on resource use could lead to differences in energy consumption in ecosystems despite similarities in species richness [74]. Alternatively, winter declines and small populations in spring may have prevented shrews from reaching numbers that compensate for the absence of mice. Previous studies have also demonstrated that shrews usually reach lower densities than mice [37].

Although our study was largely observational, the severe decline in mice densities in 2012 acted as a pseudo-removal experiment. The observed changes in the degree of overlap of the dietary niches of mice and shrews and within shrews between 2010–2011 and 2012 indicate a large shift in resource use. Our subsampling of the 2010–2011 dataset (280 mice and 231 shrews) with the number of mice and shrews represented in 2012 (21 mice and 64 shrews), provides support for this conclusion. Therefore, the differences in sample size could not explain the dietary shift observed in dusky shrews. Indeed, a shift rather than an increase in the size of the dietary niche is consistent with our observation that the numerical increase of dusky shrews only partially compensated for the changes in community energy consumption following the crash of Keen’s mice.

The crash of Keen’s mouse populations on POW followed a winter with relatively high snow accumulation. During the 2011–2012 winter, accumulation was 4× higher than in 2009–2010 and 2.5× higher than 2010–2011 (http://www.wrcc.dri.edu/cgi-bin/cliMAIN.pl?ak2227). Severe winters can reduce small mammal survival by limiting access to food and subnivean spaces, which may increase the costs of thermoregulation and predation risk [75,76]. However, snow accumulation alone cannot explain the lack of recovery of mice during the following breeding season. Also, if snow caused the crash of mice, we would expect to see the same trend in shrews. We suspect that predation, possibly facilitated by high snow accumulation, was the main cause of the demise of Keen’s mice. Our companion study of mesocarnivores revealed that abundance of both predators increased after 2010, with ermines peaking in 2011 and martens in 2012 [77]. Because martens rarely prey on shrews [78], they largely targeted mice in 2012 [77] and likely inhibited their recovery over the summer. Regular predator-prey cycles of martens, mice and long-tailed voles have been observed in Southeast Alaska [79].

Given the size of POW and recent findings that competition on large islands is low compared to medium size islands [23], our observation of the effect of Keen’s mice abundance on dusky shrews numbers and diet is significant. Schoener et al. [23] suggest that low effect size of competitive interactions on large islands is caused by spatial heterogeneity of habitat features and food availability. Nonetheless, despite its size, as a high latitude island POW is characterized by depauperate flora and fauna where heterogeneity largely stems from anthropogenic disturbance. It is likely that biotic interactions, even between unlikely competitors, may be more pronounced in depauperate systems and thus more readily detected.

The combination of temporal and spatial variation in competitive interactions we documented illustrates the importance of management practices in the TNF on community dynamics. We observed that in old-growth stands, shrews were better able to energetically compensate for the decline in mice. Although similar increases occurred in clearcuts, forest succession quickly converts them to young-growth stands. Thus, continued old-growth logging in the TNF will result in reduction in the capacity of shrews to recover from competitive pressures imposed by mice. In contrast, in pre-commercially thinned stands, shrew populations were less affected by changes in mice densities. Because shrews constitute the main prey for the endemic POW ermine during scarcity of alternative prey [77], their relative stability in thinned, open-canopy stands ensures food supply for these predators. This suggests that thinning may reverse the homogeneity resulting from clearcut logging, increase habitat quality, and help to conserve species diversity, especially of ground-dwelling small mammals. Our results support earlier studies by Carey and Wilson [80], Sullivan and Sullivan [37], and Suzuki and Hayes [81], which concluded that pre-commercial thinning or selective harvesting increased habitat quality and abundance for several small mammal species. At this juncture in the development of the new Tongass Land Management Plan, our findings may provide an incentive for accelerating the transition from old-growth clearcut logging to commercial harvest of young-growth stands.

## Supporting information

S1 Text Supplementary text and tables for the manuscript. (DOCX)

S1 Fig Comparison of Keen’s mice abundance estimates. Abundance estimates of Keen’s mice generated from the robust-design population model in Program MARK (top) and captures per 100 trap nights (100TN; bottom) in relation to the minimum number known alive (MNKA) on Prince of Wales Island, Alaska from 2010–2012.
(PNG)

S2 Fig Comparison of dusky shrew abundance estimates. Abundance estimates of dusky shrews generated from a dead-and-alive framework (top) and captures per 100 trap nights (100TN; bottom) in relation to the minimum number known alive (MNKA) on Prince of Wales Island, Alaska from 2010–2012.
(PNG)

S3 Fig Comparison of Keen’s mice density estimates. Density (number per ha) estimates of Keen’s mice generated from the robust-design population model in Program MARK in relation to estimates generated from spatially explicit capture-recapture (SECR) on Prince of Wales Island, Alaska from 2010–2012.
(PNG)

S4 Fig Niche size and overlap with kernel density estimates. 50%, 75%, and 95% contours of the isotopic niches of Keen’s mice and dusky shrews on Prince of Wales Island, Alaska 2010–2011 estimated with kernel density estimators. Mice are depicted in circles and shades of orange and shrews in triangles and purple.
(PNG)
