# Supplementary material for: Functional and numerical responses of shrews to competition vary with mouse density
Source: PLoS One. 2018 Jan 3;13(1):e0189471. doi: 10.1371/journal.pone.0189471 (PMC5752000; doi:10.1371/journal.pone.0189471)
Supplement: S1 Text — (DOCX) [file pone.0189471.s001.docx]

**Functional and numerical responses of shrews to competition vary with mouse density**

Carolyn A. Eckrich^1^, Elizabeth A. Flaherty^2^, and Merav Ben-David^1^

Supplementary materials

*Shrew trapping and mortality*

This study, in response to requests from the US Forest Service, was designed to investigate the effects of various forestry treatments on small mammals and their predators. Dusky shrews (*Sorex monticolus*) were caught incidentally in traps set for Keen’s mice (*Peromyscus keeni*) and long-tailed voles (*Microtus longicaudas*). These traps were furnished with polyester bedding to aid in thermoregulation and baited with a mix of oats, molasses and peanut butter (which was consumed by the shrews) to ensure survival of all captured small mammals. As a result of these inadvertent captures and because managed stands in the coastal rainforests of Southeast Alaska, are treacherous to navigate in daylight, and especially after dark, shrew mortality (average per grid 7; median 6) was higher than recommended by animal use and care guidelines [1]. Nonetheless, our analyses illustrate that trapping mortality had little effect on the population growth rate (see text). All mortalities were deposited in the Vertebrate Museum at the University of Wyoming (UW) Berry Center for Biodiversity Conservation (including frozen tissues), and are available to the research community through the online ARCTOS database. Given these conditions, our protocols were approved by the University of Wyoming Independent Animal Care and Use Committee (IACUC). The IACUC protocol was furnished to the journal. The incidental shrew captures provided a unique opportunity to investigate the existence of competition in a relatively simple, island ecosystem.

*Estimating the abundance of mice*

Mark-recapture data for Keen’s mice were analyzed using the robust-design population model in Program MARK [2] to generate estimates of survival and abundance. We constructed several *a priori* competing models (Table A) and selected among them based on Akaike’s information criterion values, corrected for sample size (AICc), and the significance of parameter estimates. A separate set of models was developed for each grid for the three years and 45 occasions (three primary and five secondary occasions in each sampling year). We used unequal time intervals (1 month between the three summer primary sessions and 10 months for the overwinter periods) to generate survival estimates (Table A). In all models emigration and immigration were set to zero because of data scarcity. We then calculated additional population indices (MNKA and captures per 100 trap nights [100TN]) and evaluated the relationships among them (S1 Fig).

**Table A. Population models for Keen’s mice.**

| Model | Description | Top model for |
| --- | --- | --- |
| S(monthly), p(session, year), c(session, year), N(session, year) | Monthly survival estimates, capture and re-capture probability and abundance vary by trapping session and year | 13 grids |
| S(monthly), p(session), c(session), N(session, year) | Capture and re-capture probability vary by session with no year effect | 5 grids |
| S(monthly), p(year), c(year), N(session, year) | Capture and re-capture probability vary by year | 2 grids |
| S(monthly), p(.), c(.), N(session, year) | Capture and re-capture probability are constant | 1 grid |
|  |  |  |

List of competing robust design capture-recapture models for estimating survival (S) and abundance (N) of Keen’s mice on Prince of Wales Island, Alaska, 2010-2012. Models were developed separately for each of 21 trapping girds.

*Estimating abundance of shrews*

For dusky shrews we used a dead-and-alive framework to generate abundance estimates. After removing the mortalities from the dataset for each grid, we calculated the abundance of the live animals with the Horvitz-Thompson estimator [3]. Because we had no marked individuals we used estimates of capture probability (and 95% confidence intervals) for *Sorex* shrews from a study by Otto and Roloff [4]. We treated the number of captures as minimum number known alive (MNKA). Because the live portion of the sample was unmarked, likely including several recaptures, these estimates may be biased high. Nonetheless, this bias is likely negligible because of the high mortality rate of trapped shrews (Fig 3). After producing the estimates for the live portion of the sample we added the number of mortalities to generate the total abundance estimates. We then calculated additional population indices (MNKA and 100TN) and evaluated the relationships among them (S2 Fig).

*Comparing robust design and spatially explicit capture-recapture models*

On a subset of grids we used spatially explicit capture-recapture [5] functions to estimate density and population size for Keen’s mice with the R package secr [6,7]. Density was estimated based on the location of detectors (traps) and the capture history of all known individuals within those grids. We fit several competing models and used AICc criteria for model selection. The best fitting models included a learned trap response. These models yielded higher estimates than those generated from the robust-design and mean maximum distance moved (MMDM; see main text; S3 Fig) similar to patterns observed by Gerber and Parmenter [8].

*Habitat and food availability – data reduction*

**Table B.** **Habitat variables measured within small mammal trapping grids on Prince of Wales Island, Alaska from 2010-2012.**

| Variable | Description | Unit of measurement |
| --- | --- | --- |
| LAI | Leaf-area index | As estimated from radiometry at 8 – 9 stations per grid (Eckrich et al. 2013) |
| Mushrooms | Wet weight of epigeous fungi | Grams per 1 x 1 m plot (8 or 9 per grid each year) |
| Diversity | Understory species diversity | Shannon-Weiner diversity index |
| Richness | Understory species richness | Species richness |
| Elevation | Elevation at trapping grid | In meters from a DEM |
| Blueberry | Cover of *Vaccinium* | Percent cover from a 20 m transect(8 or 9 per grid each year) |
| Herbaceous | Cover of herbaceous vegetation | Percent cover from a 20 m transect (8 or 9 per grid each year) |
| CWD | Cover of coarse woody debris | Percent cover from a 20 m transect(8 or 9 per grid each year) |
| Salmonberry | Cover of *Rubus spectabilis* | Percent cover from a 20 m transect(8 or 9 per grid each year) |
| Beetles | Average number of beetles per trap | Number from 5 pitfall traps along a 20-m transect |
| Earthworms | Wet weight of earthworms | Grams per 1 x 1 m plot (8 or 9 per grid each year) |

Understory and overstory variables were averaged to obtain stand-level values for each trapping grid. These variables (except elevation) were reduced into two factors using non-parametric multidimensional scaling techniques.

**Table C. Non-metric multidimensional scaling (NMDS) variable scores.**

| Habitat variable | NMDS1 | NMDS2 |
| --- | --- | --- |
| LAI | **0.63** | -0.05 |
| Mushrooms | 0.22 | 0.16 |
| Diversity | -0.04 | **-0.68** |
| Richness | -0.20 | 0.07 |
| Blueberry | -0.32 | -0.14 |
| Herbaceous | -0.30 | -0.25 |
| CWD | **-0.45** | **0.40** |
| Salmonberry | **-0.42** | 0.38 |
| Beetles | 0.22 | 0.06 |
| Earthworms | 0.37 | 0.19 |

Values in bold represent scores contributing the most (*r* > 0.40) to each dimension. For variable descriptions see Table S2.

The two dimensions separated the four habitat types with most of the divergence occurring on NMDS1 and some on NMDS2, especially between old growth and clearcuts (Fig 5).

*Regressive models*

**Table D. Generalized linear regressive models of dusky shrew density and estimated competition coefficients (α) on Prince of Wales Island, Alaska.**

| Year | Model | AICc | ΔAIC | *R^2^* | α |
| --- | --- | --- | --- | --- | --- |
| 2010 | SOMO_t_ = Elevation | 68.4 | 0.00 | 0.01 | - |
|  | SOMO_t_ = NMDS1 | 68.8 | 0.44 | 0.01 | - |
|  | SOMO_t_ = PEKE | 69.1 | 0.72 | 0.00 | -0.02 |
|  | SOMO_t_ = NMDS2 | 69.2 | 0.81 | 0.00 | - |
| 2011 | SOMO_t_ = PEKE | 154.8 | 0.00 | 0.20 | -0.53 |
|  | SOMO_t_ = PEKE + NMDS2 | 156.0 | 1.25 | 0.21 | -0.52 |
|  | SOMO_t_ = PEKE + NMDS1 + Elevation | 156.1 | 1.34 | 0.24 | -0.44 |
|  | SOMO_t_ = PEKE + NMDS2 + Elevation | 156.3 | 1.53 | 0.20 | -0.49 |
|  | SOMO_t_ = PEKE + NMDS1 | 156.5 | 1.78 | 0.20 | -0.50 |
| 2012 | SOMO_t_ = PEKE + Elevation | 209.0 | 0.00 | 0.14 | -1.23 |
|  | SOMO_t_ = NMDS1 + Elevation | 210.5 | 1.46 | 0.11 | - |
|  | SOMO_t_ = NMDS1 | 210.9 | 1.88 | 0.08 | - |

For all years, the dependent variable was shrew density (SOMO) at time *t*. Independent variables were Keen’s mice density at time *t*, elevation at the trapping grid, NMDS1 and NMDS2. NMDS = non-metric multidimensional scaling. Only models with considerable support (ΔAIC ≤ 2) are shown.

*Estimating niche size and overlap with kernel density estimates*

We used a two-dimensional kernel (bivariate normal) method with a normal reference bandwidth [9] to estimate niche size and overlap for mice and shrews using the R package rKIN [10]. An example of 50%, 75%, and 95% contours for both species is provided in S4 Fig.

*References*

1. Sikes RS, Bryan JA, Byman D, Danielson BJ, Eggleston J, Gannon MR, et al. 2016 Guidelines of the American Society of Mammalogists for the use of wild mammals in research and education. J Mammal. 2016;97: 663–688.

2. Cooch EG, White GC. Program MARK: A gentle introduction. 16th edition. [Internet]. 2015. Available: http://www.phidot.org/software/mark/docs/book/

3. Horvitz DG, Thompson DJ. A generalization of sampling without replacement from a finite universe. J Am Stat Assoc. 1952;47: 663–685.

4. Otto CRV, Roloff GJ. Using multiple methods to assess detection probabilities of forest-floor wildlife. J Wildl Manag. 2011;75: 423–431. doi:10.1002/jwmg.63

5. Efford MG, Borchers DL, Byrom AE. Density estimation by spatially explicit capture–recapture: likelihood-based methods. Modeling demographic processes in marked populations. Springer; 2009. pp. 255–269. Available: http://link.springer.com/chapter/10.1007/978-0-387-78151-8_11

6. Efford MG. secr: Spatially explicit capture-recapture models [Internet]. 2013. Available: http://CRAN.R-project.org/package=secr

7. R Core Team. R: A language and environment for statistical computing. [Internet]. Vienna, Austria: R Foundation for Statistical Computing; 2013. Available: http://www.R-project.org/

8. Gerber BD, Parmenter RR. Spatial capture-recapture model performance with known small-mammal densities. Ecol Appl. 2015;25: 695–705.

9. Venables WN, Ripley BD. Modern Applied Statistics with S [Internet]. New York: Springer; 2002. Available: http://link.springer.com/10.1007/978-0-387-21706-2

10. Albeke, S. E. rKIN: (Kernel) Isotope Niche Estimator Functions - rKIN. [Internet]. 2016. Available: http://github.com/salbeke/rKIN
