# Supplementary figures and images for: Functional and numerical responses of shrews to competition vary with mouse density
Source: PLoS One. 2018 Jan 3;13(1):e0189471. doi: 10.1371/journal.pone.0189471 (PMC5752000; doi:10.1371/journal.pone.0189471)

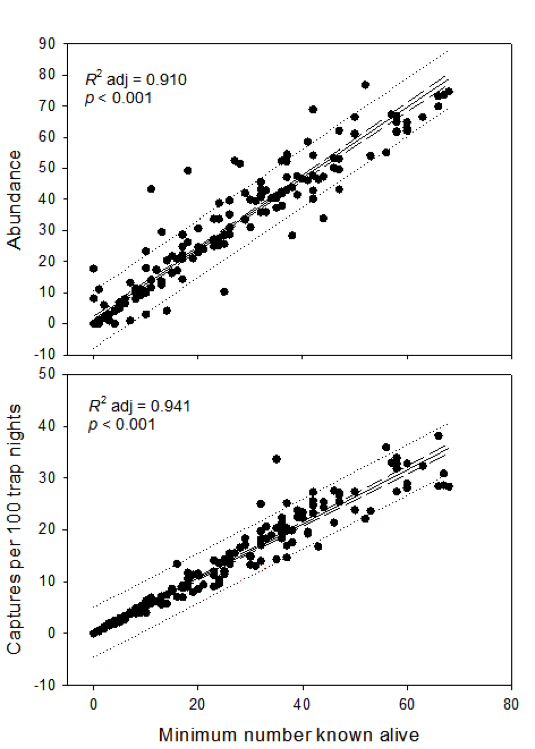

Supplement: S1 Fig — Abundance estimates of Keen’s mice generated from the robust-design population model in Program MARK (top) and captures per 100 trap nights (100TN; bottom) in relation to the minimum number known alive (MNKA) on Prince of Wales Island, Alaska from 2010–2012. (PNG) [file pone.0189471.s002.png]

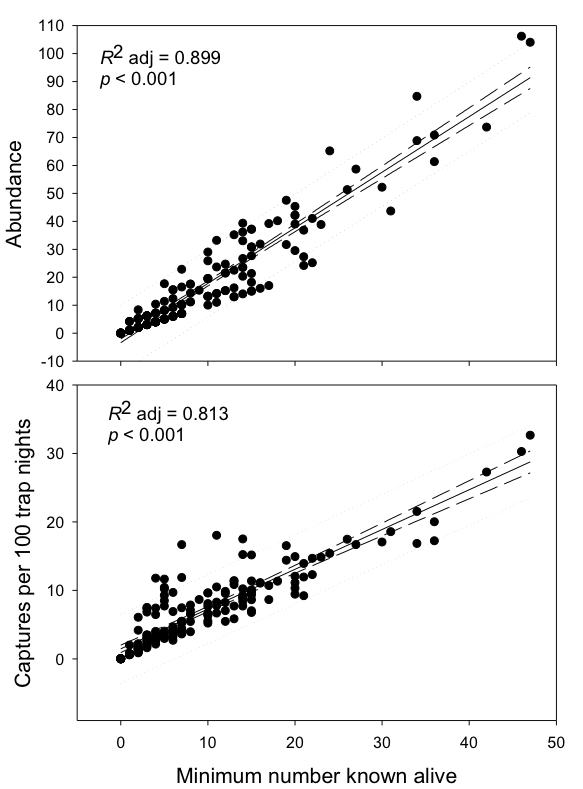

Supplement: S2 Fig — Abundance estimates of dusky shrews generated from a dead-and-alive framework (top) and captures per 100 trap nights (100TN; bottom) in relation to the minimum number known alive (MNKA) on Prince of Wales Island, Alaska from 2010–2012. (PNG) [file pone.0189471.s003.png]

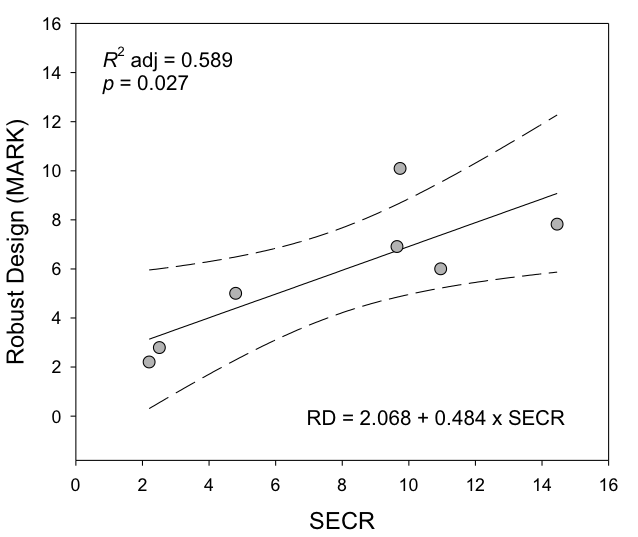

Supplement: S3 Fig — Density (number per ha) estimates of Keen’s mice generated from the robust-design population model in Program MARK in relation to estimates generated from spatially explicit capture-recapture (SECR) on Prince of Wales Island, Alaska from 2010–2012. (PNG) [file pone.0189471.s004.png]

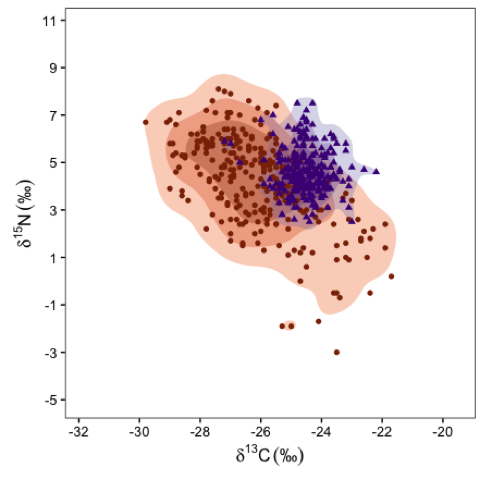

Supplement: S4 Fig — 50%, 75%, and 95% contours of the isotopic niches of Keen’s mice and dusky shrews on Prince of Wales Island, Alaska 2010–2011 estimated with kernel density estimators. Mice are depicted in circles and shades of orange and shrews in triangles and purple. (PNG) [file pone.0189471.s005.png]
